# Anxiety, depression and tinnitus: a cross-sectional study about 60 cases

**DOI:** 10.1192/j.eurpsy.2022.1186

**Published:** 2022-09-01

**Authors:** M. Karoui, A. Kchaou, G. Amri, H. Nefzi, R. Kammoun, F. Ellouz

**Affiliations:** 1 Razi hospital, Psychiatry G Department, Mannouba, Tunisia; 2 Razi Hospital, Manouba, tunis, Tunisia; 3 Razi hospital, Psychiatry G, manouba, Tunisia

**Keywords:** Anxiety, Depression, tinnitus, sleep disorders

## Abstract

**Introduction:**

Tinnitus is an auditory perception of a “phantom” nature with highly changing features. There is an established correlation between anxiety, depression, sleep disorders and tinnitus.

**Objectives:**

To evaluate the prevalence of sleep disorders and emotional disorders during tinnitus and their correlation to the severity of the symptomatology

**Methods:**

A descriptive cross-sectional study of 60 patients consulting for subjective tinnitus. For each patient we collected epidemiological data and performed an ENT and general examination, an audiometric and psychoacoustic evaluation and a psychometric evaluation. To evaluate the severity of the tinnitus we used the visual analog scale VAS and the subjective tinnitus severity test (STSS). Disability was assessed by the Tinnitus Handicap Inventory (THI). Anxiety and depression were assessed by: the Hamilton anxiety Rating scale and the Beck depression inventory.

**Results:**

The prevalence of emotional disorders was: 21.7% for depression, 48.33% for generalized anxiety disorder, 11.67% for dysthymia, 5% for agoraphobia 16.67% for panic disorder and 1.67% for social phobia. The intensity of tinnitus was correlated with more panic disorder (p=0.008). Subjective severity of tinnitus was correlated with disability (p=0.0001), awareness of tinnitus in relation to sleep duration (p=0.006) and disturbed sleep (p=0.047). Disability was correlated with subjective tinnitus severity (p=0.0001), panic disorder (p=0.0007), generalized anxiety disorder (p=0.033), and poor sleep quality (p=0.005).

**Conclusions:**

Our results emphasize the importance of emotional disorders as well as sleep disorders in chronic “tinnitus”. These disorders should be systematically investigated and eventually treated in order to optimize the management of the patients.

**Disclosure:**

No significant relationships.

